# Are Student Teachers’ Overall Expected Emotions Regarding Their Future Life as a Teacher Biased Toward Their Expected Peak Emotions?

**DOI:** 10.3389/fpsyg.2022.816456

**Published:** 2022-04-06

**Authors:** Markus Forster, Christof Kuhbandner

**Affiliations:** Department of Human Sciences, University of Regensburg, Regensburg, Germany

**Keywords:** teacher emotions, teacher education, peak-end rule, affective forecasting, affective bias

## Abstract

Having functional expected emotions regarding one’s future life as a teacher is important for student teachers to maintain their motivation to choose a career as a teacher. However, humans show several biases when judging their emotional experiences. One famous bias is the so-called peak-end effect which describes the phenomenon that overall affective judgments do not reflect the average of the involved emotional experiences but the most intense and the most recent of the involved emotional experiences. Regarding student teachers’ expected positive emotions, such a bias would be functional since their motivation to become a teacher is enhanced. However, regarding student teachers’ expected negative emotions, such a bias would be dysfunctional since their motivation to become a teacher would be decreased. The aim of the present preregistered study was to examine whether student teachers’ expected future teaching-related emotions show a peak-end effect. Student teachers viewed 14 common events that could part of a typical everyday routine of a teacher and rated their expected emotional pleasure and discomfort for each of the events. Afterward, they were asked to rate their overall expected emotional pleasure and discomfort when looking at their future professional life as a whole. Results showed that expected pleasure was much larger than expected discomfort regarding both overall, peak, and average ratings. No peak-end effect was observed for overall expected discomfort which reflected the average expected discomfort across events. By contrast, overall expected pleasure was biased toward expected peak pleasure experiences. These findings indicate that student teachers judge their expected overall affect in a functional way: realistically when dealing with negative emotions but through rose-colored glasses when dealing with positive emotions.

## Introduction

Expectations about the future emotions experienced in their later professional life as a teacher is one of the main motivational forces that motivates student teachers to invest in the acquisition of teaching-related competencies (Richardson and Watt, 2016). From a functional perspective, expecting positive emotions is advantageous since positive emotions broaden the mindset (Fredrickson, 2004) which is crucial for a deeper engagement with the to be acquired teaching-related competencies. Regarding expected negative emotions, foreseeing emotionally challenging situation in a realistic way is important to derive which competencies have to be acquired to master the challenging situations and prevent future negative emotions. However, it would be disadvantageous if one is overwhelmed by situations that are particularly emotionally challenging because this may lead to unrealistically high expected negative emotions. Such emotional dynamics may induce avoidance motivation and thus impede the acquisition of teaching-related competencies and undermine the motivation to become a good teacher (Hsieh et al., 2007).

It is known from research that when it comes to evaluating the global affective pleasure or discomfort of episodes, people’s evaluations do not reflect the average of the involved emotional experiences but are biased toward the emotional experiences that are outstanding. For instance, in the seminal study by Fredrickson and Kahneman (1993), participants were shown series of plotless short film clips with emotionally negative and positive contents of varying intensity, and they were asked to provide continuous ratings of experienced affect while watching each film. Afterward, they were asked to evaluate the overall experienced pleasure or discomfort. Results showed that the overall evaluations were well predicted by the most intense affect rating and the affect rating for the final film clip. The phenomenon that overall affective evaluations of episodes can be well predicted by the experienced peak affect intensity and the affect experienced at the end of the episode is called the peak-end effect and has been replicated several times for different types of emotional episodes, such as aversive sounds (Schreiber and Kahneman, 2000) or payment sequences (Langer et al., 2005), and even for real-life experiences such as unpleasant medical procedures (Redelmeier and Kahneman, 1996) or pain experienced during childbirth (Chajut et al., 2014; for reviews, see Fredrickson, 2000; Kahneman, 2000). In particular, in terms of real-life applications, it has been demonstrated that the peak-end effect can be successfully applied to improve people’s behavior (Redelmeier et al., 2003).

The peak-end effect may also play an important role when student teachers try to estimate the emotional pleasure and discomfort associated with their future professional life as a teacher. The everyday work of a teacher consists of several different tasks and events that trigger both positive and negative emotions of different intensities. If the evaluations of the overall expected future emotional pleasure and discomfort are influenced by the peak-end effect, student teachers’ evaluations should be biased toward their expected peak affective experiences. Regarding expected overall pleasure, this would be beneficial since the true overall pleasure would be overestimated so that the motivation to become a teacher is enhanced. However, regarding expected overall emotional discomfort, this would be dysfunctional since the true overall discomfort would be overestimated so that the motivation to become a teacher is decreased. If so, an important part of teacher education would be to help teachers be aware of such fallacies of the affective forecasting of one’s future professional life.

The aim of the present study was to examine whether student teachers’ evaluations of their expected future emotions show a peak-end effect. Student teachers were shown pictures of 14 common events that could part of a typical everyday routine of a teacher accompanied by spoken explanations. For each of the events, they were asked to rate their expected future pleasure and discomfort. After viewing all 14 events, they were asked to rate their expected pleasure and discomfort when looking at their future professional life as a whole. If student teachers evaluate their overall expected future emotional pleasure and discomfort in a more functional way, their evaluations should mirror the average pleasure and discomfort across the 14 events. If their overall expected future emotional pleasure and discomfort are influenced by the peak-end effect, their overall evaluations should be biased toward the respective expected peak affective experiences.

## Materials and Methods

### Participants

The experiment was preregistered (see https://doi.org/10.17605/OSF.IO/92ZKA) with a target sample size of 64 student teachers. Participants were recruited through advertisements at online social networks or online courses at the University of Regensburg and were paid four Euros for full participation. One participant had to be excluded due to not being a student teacher. The mean age of the remaining 63 participants (40 women and 23 men) was 21.98 years (ranging from 18 to 29 years, *SD* = 2.16), 28.6% were primary school student teachers, and 68.2% were secondary school student teachers (lower track schools: 9.5%, intermediate track schools: 19.0%, comprehensive schools: 39.7%, and others: 3.2%). The mean number of studied semesters was 4.94 semester (ranging from 2 to 7 semester, *SD* = 2.18). More detail information about the sample of participating student teachers (e.g., studies subjects) can be found at https://osf.io/h92fy/. The study was conducted in accordance with the Helsinki Declaration and the University Research Ethics Standards of the University of Regensburg. All participants provided written informed consent. In Germany, these types of psychological studies do not require ethical approval of an Ethics Committee (see https://www.dfg.de/foerderung/faq/geistes_sozialwissenschaften/).

### Material

**Figure 1:**
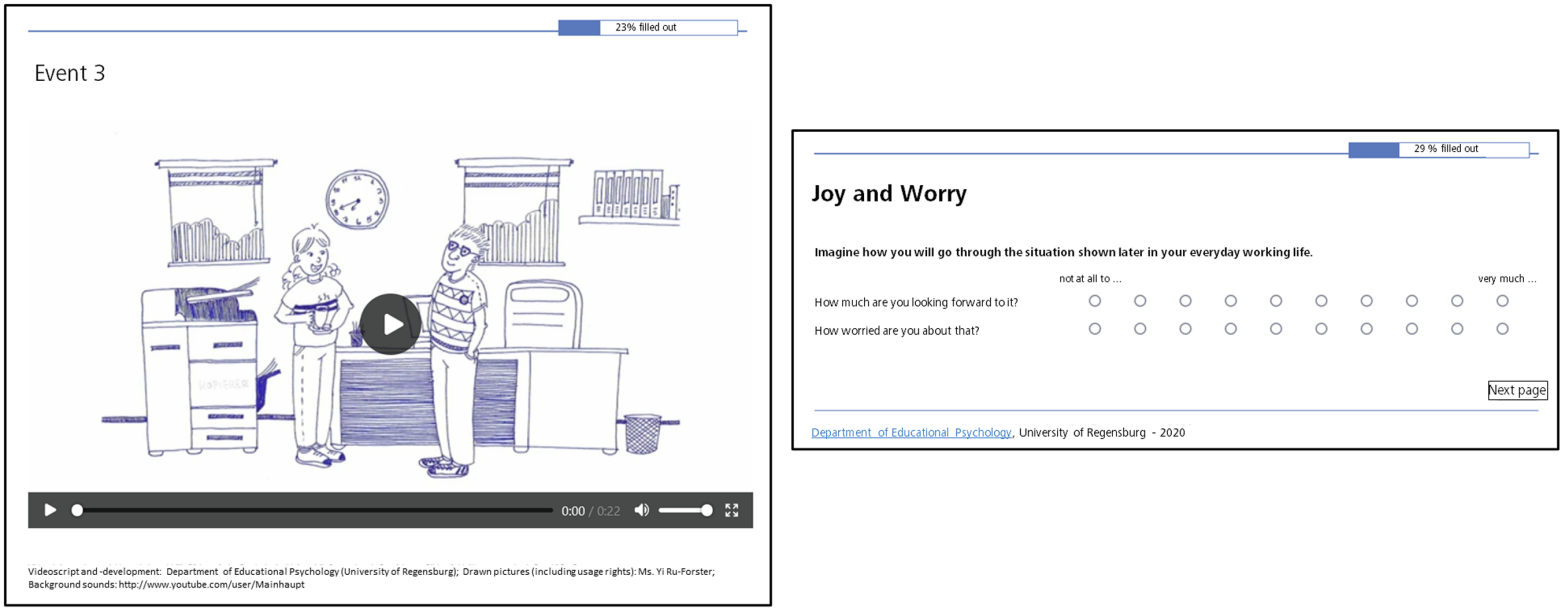
Evaluation procedure. Student teachers evaluated 14 common events that could part of a typical everyday routine of a teacher and rated their expected emotional pleasure and discomfort for each of the events. Each event was visualized by a drawing (for an example, see left side of the figure) and accompanied by an oral description. Participants were asked to imagine how they will experience the respective event in the future, and to rate how much they are looking forward to experiencing this event in the future (i.e., expected emotional pleasure) and how much they are worried about experiencing this event in the future (i.e., expected emotional discomfort) on a 10-point Likert-scale ranging from 1 (“not at all”) to 10 (“very much”; see right side of the figure). Reproduced with permission from Yi Ru-Forster, who designed the materials specifically for this study. These are available on request by the corresponding author.

Fourteen teaching-related events that are commonly part of the daily routine of the professional life of a teacher were chosen and individually presented in the form of a sequence of a possible daily routine of the professional life a teacher, starting at the morning and ending in the evening (for a description, see Table 1). Each event was illustrated by a drawing and accompanied by an oral description of the event (for an example, see Figure 1, left panel). There was a version with a female and a male teacher, and participants initially chose which version they would like to see. The selection of the events was based on a well-established tool (“SeLF”: https://www.self.mzl.lmu.de/self-starten-sie-hier/) which aims to help student teachers to get realistic insights into the different events that are part of the everyday work of a teacher and the associated requirements (Kahlert and Kriesche, 2016). This tool offers 16 short film clips about different teaching-related events that commonly occur during the working day of a teacher. The events created for the present study (drawings depicting the events, accompanied by oral descriptions) were presented pre-experimentally to various teachers who provided informal feedback, based on which the events were improved to ensure that the used event descriptions matched the real everyday working life of teachers as closely as possible. An effort was made to choose common teaching-related events that elicit both positive and negative emotions. All drawings and the durations of the oral descriptions can be found at https://osf.io/h92fy/.

**Table 1:** Description of the evaluated teaching-related events.

*Event*	*Title and description*
Event 1	**Getting up and morning routine.** The teacher introduces her/himself and begins to describe her/his daily work, describing how her/his morning routine works during the week.
Event 2	**The way to school.** Expression of thoughts on the way to school and on arriving at school. A brief characterization of welcoming pupils follows. Afterward, the teacher walks into the teachers’ room.
Event 3	**In the teachers’ room.** Working in the teachers’ room and emphasizing the weekly working hours, as well as a short conversation with another teacher colleague about awarding oral grades and an upcoming grade conference.
Event 4	**Parent-teacher talk (doorstep).** On the way to the classroom, there is an encounter with a parent who wants to talk about a pupil. The teacher now describes how important parent talks are in general and how they often proceed. However, she also emphasizes that such conversations in the corridor usually have to be postponed to another time so that the lesson time is not affected.
Event 5	**Starting a lesson.** Description of the pupils in terms of their activity in the classroom and starting the lesson with a blackboard write-up and an interactive quiz. This is followed by a brief explanation of the background of why an interactive quiz is being conducted.
Event 6	**A noisy school lesson.** Description of a restless lesson during the morning and explanation of how the teacher usually deals with it.
Event 7	**A very good school lesson.** Description of a very good lesson in which all pupils are motivated and generate many lesson inputs.
Event 8	**Planning a school trip with the class.** In the last hour of class, a school trip is planned together with the class. The school bell ends the lesson and heralds the break.
Event 9	**Conflict on the schoolyard.** During the teacher’s break supervision, an argument breaks out between two pupils, and the teacher quickly tries to clarify and settle the dispute.
Event 10	**The poster project.** In afternoon lessons, the class works on a poster project, and the posters are hung up together in the hallway. The principal walks by in the hallway and compliments the posters, even though he/she was not initially enthusiastic about the project.
Event 11	**Preparation of the next lessons.** After the last lesson, the teacher starts preparing the next lessons in the teachers’ room. The teacher says that sometimes he/she asks other colleagues if they could observe his/her lessons and give him/her feedback on the lessons to become a better teacher.
Event 12	**Development of new teaching concepts**. The teacher plans the next lessons and constantly compares the contents with the syllabus, material distribution plan, and sequence plan. In doing so, he/she describes the three main questions to be asked. This is followed by a description of the related research and copying of worksheets.
Event 13	**Correcting schoolwork.** Correcting exams and describing one’s own thoughts on the good and bad work. After 4 o’clock in the afternoon, the teacher packs up his or her things and leaves the school.
Event 14	**Parents’ phone call & future events.** The teacher has a telephone conversation with the parents at 6 p.m. Afterward, the teacher looks at his/her diary and describes everything that will happen at school next month.

### Procedure

The study was conducted online *via* SoSci Survey (Leiner, 2021). At the beginning, the participants were instructed that the aim of the study was to illustrate the tasks, expectations, and framework conditions of their future everyday life as teachers to enable a more realistic assessment of one’s future professional practice. The following instruction was given (original in German):

“In the following, we will show you scenes with the aim to illustrate the tasks, expectations, and framework conditions of the everyday professional life of teachers in order to enable you to make a more realistic assessment of your future professional practice. A possible daily routine with situations from the everyday working life of a teacher, from getting up to going to bed, will be shown. After each scene, we will ask you for a personal evaluation in which we encourage you to think about your expected future joy and worry regarding the respective situation.”

Then, the sequence of the 14 events was shown. After each of the events, participants were asked to imagine how they will experience the respective event in the future, and to rate how much they are looking forward to experiencing this event in the future (i.e., expected emotional pleasure) and how much they are worried about experiencing this event in the future (i.e., expected emotional discomfort) on a 10-point Likert-scale ranging from 1 (“not at all”) to 10 (“very much”; for an illustration, see Figure 1, right panel). After providing their ratings, the participants started the presentation of the next event by a mouse click.

After the presentation and evaluation of all 14 events, the participants were asked to imagine their future professional life as a teacher as a whole, and to rate how much they are looking forward to it (i.e., overall expected emotional pleasure) and how much they are worried about it (i.e., overall expected emotional discomfort) using the same 10-point Likert scales as described above. The exact instruction was (original in German):

“You have now imagined how the typical day-to-day routine of a teacher may look like. If you finally look at your future professional life as a teacher as a whole: How much are you looking forward to it [worried about it]?”

## Results

### Affective Ratings

**Figure 2:**
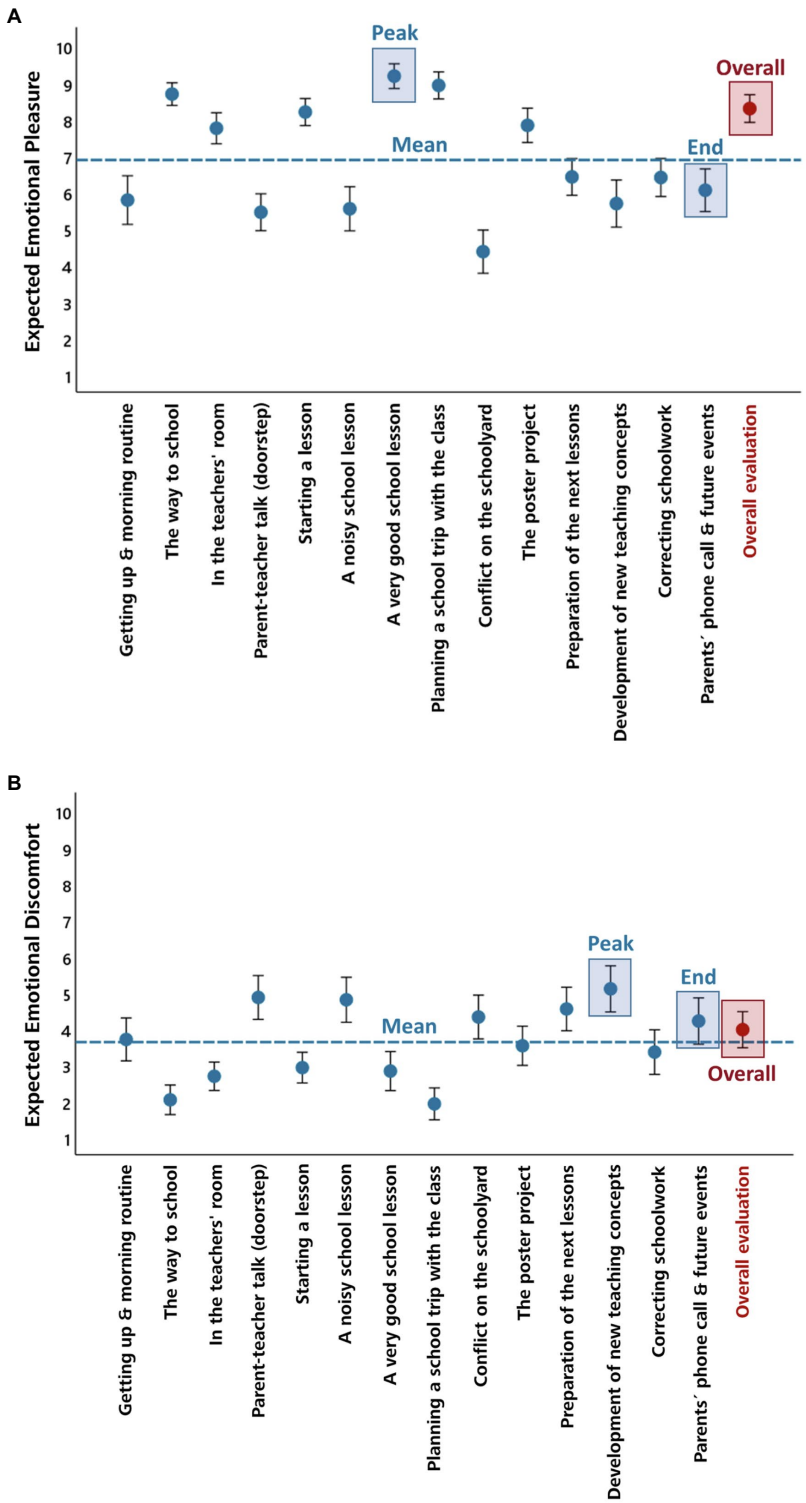
Affective ratings. The mean expected emotional pleasure **(A)** and mean expected emotional discomfort **(B)** for each of the 14 evaluated teaching-related events (blue colors) and for the future professional life as a teacher as whole (red color) are shown. The dashed horizontal lines show the average affective experience across events, the underlaid squares show the event with the peak expected affect and the event evaluated at the end of the sequence. Error bars represent 95% CIs.

The expected emotional pleasure and discomfort rated for each of the future teaching-related events and for the future professional life as a teacher as whole is shown in Figure 2A (expected emotional pleasure) and Figure 2B (expected emotional discomfort). The dashed horizontal lines show the average affective ratings across events, the underlaid squares show the event with the expected peak affect and the event evaluated at the end of the sequence.

The three teaching-related events that were associated with the highest expected emotional pleasure were “A very good lesson” (*M* = 9.16, *SD* = 1.35), “Planning a school trip with the class” (*M* = 8.90, *SD* = 1.47), and “The way to school” (*M* = 8.67, *SD* = 1.23). The three teaching-related events that were associated with the highest emotional discomfort were “Development of new teaching concepts” (*M* = 5.08, *SD* = 2.53), “Parent-teacher talk (doorstep)” (*M* = 4.84, *SD* = 2.40), and “A noisy school lesson” (*M* = 4.78, *SD* = 2.47).

For the expected peak affective experience, expected emotional pleasure was much higher than expected emotional discomfort (emotional pleasure: *M* = 9.71, *SD* = 0.63; emotional discomfort: *M* = 6.97, *SD* = 2.18), *t*(62) = 9.98, *p* < 0.001, *d* = 1.26. For the mean across events, expected emotional pleasure was much higher than expected emotional discomfort as well (emotional pleasure: *M* = 6.86, *SD* = 1.04; emotional discomfort: *M* = 3.61, *SD* = 1.31), *t*(62) = 13.79, *p* < 0.001, *d* = 1.74. For the expected overall emotional pleasure and discomfort, expected emotional pleasure was much higher than expected emotional discomfort as well (emotional pleasure: *M* = 8.27, *SD* = 1.51; emotional discomfort: *M* = 3.95, *SD* = 1.98), *t*(62) = 12.10, *p* < 0.001, *d* = 1.52.

### Peak-End Effect

As can be seen in Figures 2A,B, overall expected affect was closer to the expected peak affective experience for emotional pleasure (*M*_Difference Peak-Overall_ = 1.44, *SD* = 1.49) compared to emotional discomfort (*M*_Difference Peak-Overall_ = 3.02, *SD* = 1.79), *t*(62) = −5.03, *p* < 0.001, *d* = 0.63. By contrast, overall expected affect was closer to the mean across the evaluated events and closer to the affect expected for the event evaluated at the end of the sequence for emotional discomfort (*M*_Difference Overall-Mean_ = 0.34, *SD* = 1.28; *M*_Difference Overall-End_ = −0.24, *SD* = 2.17) compared to emotional pleasure (*M*_Difference Overall-Mean_ = 1.41, *SD* = 1.18; *M*_Difference Overall-End_ = 2.24, *SD* = 2.09), *t*(62) = 4.31, *p* < 0.001, *d* = 0.54, and *t*(62) = 5.30, *p* < 0.001, *d* = 0.67. These findings indicate that overall expected emotional pleasure was biased toward expected peak pleasure, whereas overall expected emotional discomfort was mainly determined by the average expected discomfort across the evaluated events.

To further explore the existence of a peak-end effect, a regression analysis was performed to analyze how much variance in overall expected affect was explained by the mean across the evaluated events, by the expected peak affective experience, and by the event evaluated at the end of the sequence. First, Pearson correlation coefficients were calculated, which are shown in Table 2. For expected emotional discomfort, overall expected discomfort was correlated with the mean discomfort across events, the peak discomfort, the end discomfort, and the mean between peak and end discomfort, and was most highly correlated with the mean discomfort across events.

**Table 2:** Rating results: correlations and descriptive statistics.

	1	2	3	4	5	6	7	8	9	10
1. Overall discomfort		0.77^**^	0.63^**^	0.57^**^	0.67^**^	−0.31*	−0.24	0.04	−0.11	−0.09
2. Mean discomfort			0.78^**^	0.61^**^	0.78^**^	−0.18	−0.26*	−0.02	−0.14	−0.14
3. Peak discomfort				0.57^**^	0.87^**^	−0.13	−0.30*	0.15	−0.19	−0.14
4. End discomfort					0.90^**^	−0.25	−0.30*	0.09	−0.49^**^	−0.42^**^
5. Peak-end discomfort						−0.21	−0.34^**^	0.13	−0.40^**^	−0.33^**^
6. Overall pleasure							0.62^**^	0.24	0.47^**^	0.49^**^
7. Mean pleasure								0.43^**^	0.59^**^	0.65^**^
8. Peak pleasure									0.20	0.44^**^
9. End pleasure										0.97^**^
10. Peak-end pleasure										
*M*	3.95	3.61	6.97	4.19	5.58	8.27	6.86	9.71	6.03	7.87
*SD*	1.98	1.31	2.18	2.55	2.09	1.51	1.04	0.63	2.32	1.26

* indicates *p* < 0.05;

** indicates *p* < 0.01 and *p* values are not corrected for multiple testing. Significant correlations are printed in bold.

For expected emotional pleasure, overall expected pleasure was most highly correlated with the mean across the evaluated events as well. However, surprisingly, overall expected pleasure was not correlated with the expected peak pleasure experience. This finding must be interpreted taking into account the fact that almost all participants had rated the maximum value for their expected peak pleasure experience (79.4% of participants) so that expected peak pleasure showed hardly any variance across participants (*SD* = 0.63). The consequence is that even if overall expected pleasure was highly determined by the expected peak pleasure, both values would only weakly be correlated because the occurrence of correlations presupposes that variables show a certain amount of variation.

Due to the small variance of expected peak pleasure across participants, the results of a regression analysis cannot be validly interpreted. Because of this, a regression analysis was performed only for expected emotional discomfort to compare the contributions of expected mean discomfort across events, expected peak discomfort, and end discomfort to explain the variance in expected overall emotional discomfort. The results are shown in Table 3. Only the expected mean discomfort across events turned out to be a significant predictor, with the model explaining 61.1% of the variance, indicating that overall expected discomfort was strongly determined by the mean expected discomfort across the evaluated events.

**Table 3:** Results of regression analysis predicting level of expected overall emotional discomfort from mean expected discomfort, peak expected discomfort, and end expected discomfort.

Parameter	*B*	95% CI		*SE B*	*β*	*t*	*p*
		*LL*	*UL*				
*Mean expected discomfort*	0.99	0.57	1.41	0.21	0.65	4.72	<0.001
*Peak expected discomfort*	0.04	−0.20	0.28	0.12	0.04	0.31	0.76
*End expected discomfort*	0.11	−0.05	0.27	0.08	0.14	1.36	0.18

CI, confidence interval; LL, lower limit; UL, upper limit; β, standardized regression coefficient; *t*, *t* value; *p*, probability of committing a Type-I-Error; and *p* values are not corrected for multiple testing.

## Discussion

When evaluating the overall global affective pleasure or discomfort of episodes, people’s evaluations often do not reflect the average of the involved emotional experiences but the most intense and the most recent of the involved emotional experiences (for reviews, see Fredrickson, 2000; Kahneman, 2000). The present study examined whether such a peak-end effect occurs also when student teachers predict their affective experiences elicited in their future professional life as a teacher. The results showed that student teachers’ evaluations of the overall expected affect are strongly biased toward the expected peak affect for emotional pleasure but only weakly for emotional discomfort. In the latter case, the overall expected discomfort was mainly determined by the average expected discomfort across the evaluated teaching-related events.

Regarding previous research on the peak-end effect, the present findings demonstrate that overall affective evaluations of episodes are not only biased toward peak affective experiences when people evaluate past episodes but also when people predict the affective experiences elicited by future episodes. Such a finding is interesting because previous research has shown that the biases found for remembered affective experiences are not necessarily also found for predicted future affective experiences (Levine et al., 2018). However, the present results further indicate that a bias toward expected peak affective experiences when evaluating the overall affective consequences of future episodes seems not to be a general effect found for any type of affective forecasting. A bias toward the peak was only found when evaluating the expected overall pleasure but not when evaluating the expected overall discomfort.

Such a finding is in line with previous findings showing that the peak-end effect is not a general phenomenon that occurs for any type of episodes. For instance, it has been shown that for global affective evaluations of the previous day (Miron-Shatz, 2009) or for global affective evaluations of musical pieces (Schäfer et al., 2014); the average intensity across experienced moments can play even a larger role than the peak and end experiences. To explain such findings, it has been suggested the heterogeneity of the involved experiences may play a role: when a to be evaluated episode consists of more complex and heterogeneous experiences, the overall expected affect may reflect more the average across experiences than peak and end experiences (Kemp et al., 2008; Strijbosch et al., 2019).

Since the teaching-related events evaluated in the present study are also complex and heterogeneous events, this may explain the absence of a peak-end effect for expected emotional discomfort. In particular, this may also provide an explanation for the finding that a peak effect was found only for expected emotional pleasure but not for emotional discomfort. As shown in several studies, negative affective experiences are more heterogeneous than positive affective experiences (for a review, see Unkelbach et al., 2019), a phenomenon that has been made famous by the opening line of Leo Tolstoy’s Anna Karenina: “All happy families are all alike; each unhappy family is unhappy in its own way.” Accordingly, due to the higher degree of heterogeneity, the overall evaluation of negative affective experiences may be less affected by the peak-end effect than the overall evaluation of positive affective experiences.

Regarding the occurrence of an end effect, it is important to note that the sequence of the evaluated events followed the order of how the respective events occur during a typical day of the professional life of a teacher. Consequently, all participants evaluated the events in the same order, and the last event represented the event that occurs at the end of the daily routine. Since it is assumed that overall evaluations of episodes are biased toward peak and end events because these events are outstanding, this may explain why no end effect was observed in the present study. Participants may have mentally classified the last event as the event that naturally ends the working day of a teacher, which may have led to the experience that this event is not outstanding.

The reason for presenting the to be evaluated events in the order of how the respective events occur during a typical day was that the present study focused on the question of whether a peak-end effect occurs when student teachers imagine the emotional pleasure and discomfort associated with their future everyday working life as a teacher. Accordingly, it remains to be shown whether the present findings can be generalized to situations where students imagine emotional events that are not commonly occurring in their later everyday working life. Examining this issue would be an interesting avenue for future research.

From an applied perspective, the fact that when student teachers assess the emotional pleasure and discomfort associated with their future professional life only overall expected pleasure is biased toward the expected peak affect but not overall expected discomfort is beneficial for the motivation to become a teacher. Regarding expected overall emotional pleasure, a bias toward the expected peak pleasure is functional because the average pleasure is overestimated so that the motivation to become a teacher is enhanced. Regarding expected overall emotional discomfort, the absence of a bias toward the expected peak discomfort is functional because the average displeasure is not overestimated and thus the motivation to become a teacher not decreased.

However, despite the obvious motivational functionality of student teachers’ evaluations of their future teaching-related affective experiences, it is important to note that the expected overall pleasure overestimated the average pleasure across the events that are part of the daily routine of the later professional life of a teacher. Thus, to avoid later disappointments, it seems nevertheless important to help student teachers to develop a realistic view of all parts of their later professional life as a teacher, and to develop competencies that maximize the future positive affect and minimize the future negative affect.

Beyond the actual research question of the present study, the findings are also interesting in terms of which parts of the professional life of a teacher are expected by student teachers to be emotionally rewarding and which are expected to be emotionally challenging. Regarding emotionally challenging events, the highest rated events were development of new teaching concepts, parent-teacher talk, a noisy school lesson, preparation of the next lessons, conflict on the schoolyard, and parents’ phone call, which closely resemble previous findings indicating that classroom discipline, motivating students, dealing with individual differences, relationship with parents, organization of class work, and dealing with problems of individual students are among the most important problems mentioned by teachers at the beginning of their profession (Veenman, 1984). Regarding emotionally rewarding events, the highest rated events were a very good school lesson, planning a school trip with the class, the way to school, starting a lesson, the poster project, in the teachers’ room, which mainly mirrors the main motive in choosing a career as a teacher (Rothland, 2011), namely, the joy of working with children and young people.

The results also show that the judgments of emotional pleasure and discomfort associated with a specific event vary across participants. Such interindividual differences in expected future emotions may stem from different pre-existing emotional experiences with the respective event. Furthermore, expected future emotions of student teachers’ may additionally vary as a function of the extent to which a future teaching-related event, and the coping with the related requirements, was part of the teacher education. With regard to the present analysis of the occurrence of a peak-end effect, such interindividual differences do not introduce any problematic bias since it does not matter which of the judged events is experiences as the one that elicits the most intense emotions. However, when interpreting the intensity of emotions associated with specific events, pre-existing experiences have to be taken into account.

Regarding interindividual differences, previous research has shown that states of anxiety or depression can modulate affective ratings (e.g., Teismann et al., 2020). Accordingly, one potential limitation of the present study is that participants’ states of anxiety or depression were not assessed. However, although it has been shown that students sometimes experience higher levels of anxiety and depression (e.g., Ramón-Arbués et al., 2020), the reasons for such mental problems are typically not the later job requirements, but instead the experience of high demands during study and performance pressure (Fabricius et al., 2019). Thus, since the present research question focused on affective experiences associated with events that are part of their future work, mental problems stemming from study-related aspects such as high study demands may have played only a minor role in the present study.

Both for expected peak affective experience, for the mean across events, and for the expected overall affect, ratings for emotional pleasure were higher than for emotional discomfort. Such a pattern mirrors the typical finding in studies examining affective ratings that affective ratings are higher for positive than for negative experiences. A prominent example is the ratings of positive and negative affect using the PANAS scales. For instance, in the validation study (Watson et al., 1988), participants provided higher affect ratings for positive than for negative affect items regardless of the used time frame. Similarly, regarding ratings in the domain of emotional wellbeing, a finding that is found throughout the world is that most people’s affect is primarily pleasant (e.g., Diener and Diener, 1996). Regarding possible explanations, it has been speculated, for instance, that the internal neutral set point is shifted toward the positive range of affective scales (Headey and Wearing, 1992), or that people’s emotional assessments are biased toward positive evaluations due to motivational reasons because positive affect energizes approach tendencies which are important for long-term survival (Diener and Diener, 1996).

In conclusion, the present study demonstrates that student teachers’ expected affective experiences regarding their future life as a teacher are biased toward expected peak emotions, but only for expected pleasure and not for expected discomfort. For expected discomfort, the expected overall affect reflected the average expected discomfort across the evaluated teaching-related events. These findings indicate that student teachers seem to judge their expected overall affect in a functional way: realistically when dealing with negative affective experiences but through rose-colored glasses when dealing with positive affective experiences.

## Data Availability Statement

The datasets presented in this study can be found in online repositories. The names of the repository/repositories and accession number(s) can be found at: https://osf.io/h92fy/.

## Ethics Statement

Ethical review and approval was not required for the study on human participants in accordance with the local legislation and institutional requirements. The patients/participants provided their written informed consent to participate in this study.

## Author Contributions

MF and CK developed the research idea and drafted the manuscript. MF designed the study and analyzed the data. All authors contributed to the article and approved the submitted version.

## Funding

The project which this article is part of is supported by the federal and state governments as part of the joint “Qualitätsoffensive Lehrerbildung” (Teacher Education Quality Offensive), which is funded from the Federal Ministry of Education and Research under the grant number 01JA1812. The authors are responsible for the content of this publication.

## Conflict of Interest

The authors declare that the research was conducted in the absence of any commercial or financial relationships that could be construed as a potential conflict of interest.

## Publisher’s Note

All claims expressed in this article are solely those of the authors and do not necessarily represent those of their affiliated organizations, or those of the publisher, the editors and the reviewers. Any product that may be evaluated in this article, or claim that may be made by its manufacturer, is not guaranteed or endorsed by the publisher.
